# FMRP Controls Neuronal Architecture and Synaptic Content of NMDA Receptors in Cultured Hippocampal Neurons

**DOI:** 10.1007/s12031-025-02325-8

**Published:** 2025-04-02

**Authors:** Elisa Corti, Carlos B. Duarte

**Affiliations:** 1 CNC- Center for Neuroscience and Cell Biology, University of Coimbra, Coimbra, Portugal; 2 CIBB- Center for Innovative Biomedicine and Biotechnology, University of Coimbra, Coimbra, Portugal; 3 Institute for Interdisciplinary Research (IIIUC), University of Coimbra, Coimbra, Portugal; 4 Doctoral Programme in Experimental Biology and Biomedicine (PDBEB), Institute for Interdisciplinary Research, University of Coimbra, Coimbra, Portugal; 5 Department of Life Sciences, University of Coimbra, Coimbra, Portugal

**Keywords:** Neuronal excitability, Axonal initial segment, GluN2A, GluN2B

## Abstract

Fragile X syndrome is the most common inherited form of intellectual disability and is caused by the transcriptional silencing of the *Fmr1* gene and the lack of fragile X messenger ribonucleoprotein (FMRP). FMRP is an RNA-binding protein that regulates the synthesis of synaptic proteins which are essential for proper brain function. Although circuit hyperexcitability is a hallmark of fragile X syndrome (FXS), the cell-autonomous effects of FMRP deficiency remain poorly understood. In this work, we investigated the functional consequences of the absence of FMRP on neuronal morphology and on ionotropic glutamate receptor surface distribution, using primary cultures of mice hippocampal neurons isolated from wild-type (WT) and *Fmr1* knock-out (KO) pups. MAP2 staining of *Fmr1* KO neurons showed a decrease in total dendritic length and complexity of the dendritic tree, accompanied by an increase in soma size compared to WT neurons. Moreover, immunolabelling of surface glutamate receptors performed under non-permeabilising conditions showed that *Fmr1* KO neurons presented a higher content of synaptic surface GluN2A and a lower content of GluN2B subunits of NMDA receptors, while GluA1 and GluA2 distribution remained unchanged. Finally, multielectrode array data showed that *Fmr1* KO neurons presented reduced spontaneous activity compared to control neurons. These data support the hypothesis that at the cellular level, *Fmr1* KO hippocampal neurons are less excitable due to altered input processing, driven by structural defects and altered GluN2A expression in the synaptic plasma membrane.

**Supplementary Information:**

The online version contains supplementary material available at 10.1007/s12031-025-02325-8.

## Introduction

Fragile X syndrome (FXS) is an X-linked neurodevelopmental disorder (Berger and Weaver 1985; Martin and Bell 1943) and represents the most common inherited form of intellectual disability (ID), affecting 1:4000 males and 1:8000 females (Peprah 2012). Despite being a heterogeneous condition, the majority of FXS patients present symptoms such as a lower IQ, stereotyped and aggressive behaviours, avoidant eye gaze, sleep disorders, anxiety and emotional lability (Protic et al. 2022). FXS is caused by the lack of fragile X messenger ribonucleoprotein (FMRP), either due to an expansion of a CGG triplet (> 200 repetitions) in the 5’-untranslated region (UTR) of the *Fmr1* gene that triggers DNA methylation and gene silencing (Sutcliffe et al. 1992; Verkerk et al. 1991) or due to missense mutations that impair the functionality of FMRP (De Boulle et al. 1993; Myrick et al. 2014). FMRP is an RNA-binding protein (Ashley et al. 1993) that plays a pivotal role in the transport, stability and ribosomal accessibility of a diverse array of mRNAs. Approximatively 4% of the brain mRNAs interact with FMRP (Ashley et al. 1993) that regulates local synthesis of proteins fundamental for a proper neuronal development and synaptic functionality. For example, the mRNAs that encode for microtubule-associated protein 1A (MAP1A) and 1B (MAP1B), postsynaptic density protein 95 (PSD95), Ca^2+^- and calmodulin-dependent protein kinase II alpha (CamKIIα) and the GluN2A and GluN2B subunits of N-methyl-D-aspartate receptors (NMDAR) are FMRP targets (Darnell et al. 2011).

Insights about the function of FMRP have been gathered from studies using *Fmr1* KO animals that recapitulate the behavioural phenotypes observed in patients (e.g. defects in learning, cognition, memory and sociability) (Melancia and Trezza 2018). Among the most reported consequences of the lack of FMRP, there is an increased density of dendritic spines which present an immature morphology (despite this phenotype being age- and brain-region dependent) (He and Portera-Cailliau 2013) and circuit hyperexcitability (Liu et al. 2021). Circuit hyperexcitability in *Fmr1* KO animals may arise from (i) an atypical activity-dependent synaptic pruning of excitatory synapses during development, (ii) a decrease of inhibitory tone onto principal neurons or (iii) modification of active and passive properties of principal neurons. Indeed, neurons from *Fmr1* KO animals show a larger spine density and altered spine dynamics, probably due to a defective microglia-dependent pruning during development (Jawaid et al. 2018). Moreover, *Fmr1* KO mice exhibit decreased expression of GABA_A_ receptor subunits (D'Hulst et al. 2006; Gantois et al. 2006), in accordance with recent evidence showing that FMRP modulates tonic inhibition by interacting with the subunit α5 of GABA_A_ receptors in the dentate gyrus (Deng et al. 2022). Additionally, FMRP modulates the expression and function of a variety of ligand-gated ion channels, such as NMDAR, and voltage-gated Na^+^, K^+^ and Ca^++^ channels (reviewed in Deng and Klyachko (2021)). In this scenario, it is difficult to pinpoint which phenotype(s) are a direct consequence of the FMRP deficiency and which are compensatory changes. 

Excitability is the capability of neurons to fire action potentials when depolarised beyond a certain threshold following stimulation of excitatory synapses. Glutamate is the major excitatory neurotransmitter in the brain, acting mainly through stimulation of α-amino-3-hydroxy-5-methyl-4-isoxazolepropionic acid receptors (AMPAR) and NMDAR present on the postsynaptic membrane within dendritic spines. AMPAR and NMDAR are ligand-gated cationic channels that upon opening produce a local depolarisation of the postsynaptic membrane (Corti and Duarte 2023; Hansen et al. 2021). As these excitatory depolarising signals travel towards the soma, they are integrated with inhibitory hyperpolarizing signals, coming from inhibitory synapses located on the dendritic shaft or on the cell body of neurons. Whenever the integrated signal produces a net depolarisation that is strong enough to open voltage-gated Na^+^ channels, an action potential is generated and propagated across the axon. Voltage-gated Na^+^ channels are accumulated in the proximal region of the axon in a structure named axonal initial segment (AIS), which separates the axon from the somatodendritic compartment (Chavlis and Poirazi 2022; Hille 1978; Magee 2000).

Neuronal excitability is shaped by the combination of passive properties and active properties of a neuron. Passive properties allow the propagation of electric signals without the use of voltage- or ligand-gated ionic channels and are influenced by the amount of neuronal plasma membrane, namely the area of the soma and the complexity of the dendritic tree. Instead, active properties are a direct expression of the abundance of ligand- and voltage-gated ion channels. In this work, we hypothesise that FMRP directly or indirectly regulates neuronal excitability by acting on both active and passive properties of hippocampal neurons. In particular, we used low-density primary cultures to investigate the cell-autonomous consequences of the lack of FMRP on passive and active properties of hippocampal pyramidal neurons, with a main focus on neuronal morphology, synaptic density and membrane content of glutamate receptors.

## Materials and Methods

### Mouse Model and Genotyping

*Fmr1* knock-out (KO) females (B6.129P2-Fmr1^tm1Cgr^/J) were purchased from Jackson laboratory and crossed with C57BL/6J male mice to obtain heterozygous females for the *Fmr1* gene. To obtain wild-type (WT) and *Fmr1* KO male pups, *Fmr1* heterozygous females were crossed with WT males. Two to five littermates, sex-matched animals were kept in each cage, with food and water ad libitum and a 12-h dark/light cycle. Animals were handled according to Portuguese Law (DL 113/2013) and the European Community Guidelines (Directive, 2010/63/EU) on the protection of animals for scientific experimentation.

The genomic DNA to perform the genotyping of the animals was extracted from a piece of tail in case of pups or from an ear punch in case of adult animals, using the HOTSHOT method (Truett et al. 2000). Briefly, each piece of tissue was placed in a tube and incubated in an alkaline solution (25 mM NaOH, 0.2 mM EDTA, pH 12) for 30 min at 95 °C. Samples were allowed to cool down to 4 °C and successively an equal volume of the neutralising reagent (40 mM Tris–HCl, pH 5) was added to each tube. Tubes were briefly centrifuged to allow the undigested tissue to sink to the bottom. Polymerase chain reaction (PCR) was performed using the NZYTaq II 2 × Green Master Mix (NZYTech) according to manufacturer’s instruction, in a final volume of 15 µl. The following primers were used: 5’-TGT GAT AGA ATA TGC AGC ATG TGA-3’ (wild-type forward oIMR6734, Jackson Laboratory), 5’-CAC GAG ACT AGT GAG ACG TG-3’ (mutant forward oIMR2060, Jackson Laboratory) and 5’-CTT CTG GCA CCT CCA GCT T-3’ (common reverse oIMR6735, Jackson Laboratory). The following cycling temperatures and time were used: the initial denaturation was performed at 94 °C for 5 min, then denaturation at 94 °C for 30 s, annealing at 57 °C for 35 s and elongation at 72 °C for 35 s were repeated for 230 cycles, followed by a final elongation step at 72 °C for 5 min. The products of the PCR were then loaded on a 1% agarose gel in TAE buffer (NZYTech) containing GreenSafe (1:10,000, NZYTech) and separated by electrophoresis (100 V for 30 min). The fragment amplified from WT animals was 131 base pair (bp) long; the fragment amplified by Fmr1 KO animals was _~_400 bp long, while heterozygous females presented both fragments.

### Primary Cultures of Hippocampal Neurons

Primary cultures of hippocampal neurons were prepared from postnatal day 0 or 1 (P0-P1) WT or *Fmr1* KO male pups, as described in Nerli et al. (2020), with slight modifications. Briefly, mice were decapitated and both hippocampi were dissected in ice-cold Hank’s balanced salt solution (HBSS: 5.36 mM KCl, 0.44 mM KH_2_PO_4_, 137 mM NaCl, 4.16 mM NaHCO_3_, 0.34 mM Na_2_HPO_4_·2H_2_O, 5 mM glucose, 1 mM sodium pyruvate, 10 mM HEPES and 0.001% phenol red, pH 7.2). The hippocampi were pooled together according to the genotype and digested with 0.25% Trypsin (GIBCO) diluted 1:5 in HBSS, to reach a final concentration of 0.05%, for 11 min at 37 °C. Then, the enzyme was blocked by adding an equal volume of HBSS + 10% foetal bovine serum (FBS, GIBCO), and the tissue was centrifuged at 11 × g for 5 min at room temperature (RT). The tissue was then mechanically dissociated in Neurobasal medium-A (NBM-A; GIBCO) supplemented with 0.12 mg/ml gentamicin (GIBCO), 2% B27 (GIBCO) and 0.5 mM l-glutamine. Approximatively 800 µl of supplemented NBM-A per pup was used, to obtain a cell suspension of the adequate concentration. Alive cells were counted using the dye exclusion method with trypan blue (GIBCO) and a Neubauer chamber.

For immunocytochemistry experiments, 11 × 10^3^ cells/cm^2^ were plated on poly-d-lysine-coated glass coverslips. Two hours later, coverslips were flipped on a feeder layer of rat glial cells in NBM-A (GIBCO) supplemented with 0.12 mg/ml gentamicin (GIBCO), 2% B27 (GIBCO) and 0.5 mM l-glutamine. Neurons grew facing the glial cells but were kept separated from the glial cells thanks to paraffin dots on the neuronal side of the coverslips.

For multielectrode array experiments, poly-d-lysine-coated CytoView MEA 24 multiwells (Axion BioSystems) were used. To obtain the proper number of cells in the required volume, cells were centrifuged (8 × g for 3 min, RT) and resuspended in NBM-A (GIBCO) supplemented with 0.12 mg/ml gentamicin (GIBCO), 2% B27 (GIBCO), 0.5 mM l-glutamine and 10 µg/ml laminin (Sigma-Aldrich), when necessary. 25 × 10^3^ cells in 10 µl were plated in the centre of each well, in correspondence of the 16 electrodes array, and 2 h later 500 µl of NBM-A (GIBCO) supplemented with 0.12 mg/ml gentamicin (GIBCO), 2% B27 (GIBCO) and 0.5 mM l-glutamine were added to each well.

Cultures were maintained in a humidified incubator at 37 °C, with an atmosphere of 95% air/5% CO_2_ for 2 weeks. A third of the initial volume of NBM-A (GIBCO) supplemented with 0.12 mg/ml gentamicin (GIBCO), 2% B27 (GIBCO) and 0.5 mM l-glutamine was added to the wells on day in vitro (DIV) 7. To prevent excessive growth of glial cells, the culture medium was supplemented with 10 µM 5-fluoro-2-deoxy-uridine (Sigma-Aldrich) on DIV2.5–3.

### Immunocytochemistry

Labelling of surface glutamate receptors was performed as described in Afonso et al., (2019) and Mele et al. (2021). Briefly, we used subunit-specific antibodies (see supplementary table I) against epitopes present in the extracellular region of the receptors. Live neurons were incubated with the antibodies diluted 1:100 in conditioned medium, for 10 min at room temperature (RT) and quickly washed in phosphate saline buffer (PBS: 137 mM NaCl, 2.7 mM KCl, 1.8 mM KH_2_PO_4_, 10 mM Na_2_HPO_4_·2H_2_O, pH 7.4). Cells were fixed in 4% paraformaldehyde (Acros Organic)—4% sucrose in PBS for 15 min at RT and washed three times with PBS. Cells were then permeabilised with ice-cold 0.3% (v/v) Triton-X-100 in PBS for 5 min and incubated with 10% (w/v) bovine serum albumin (BSA) (NZYTech) in PBS for 1 h at RT to block nonspecific epitopes. The preparations were then incubated with primary antibodies targeting intracellular proteins (see supplementary table I) diluted in 3% (w/v) BSA in PBS, overnight at 4 °C, and washed three times with PBS on the following day. Then, cells were incubated with the appropriate secondary antibodies (see supplementary table I) for 1 h at room temperature, washed three times with PBS and mounted on microscope slides (Avantor) using fluorescence mounting medium (DAKO).

### Image Acquisition and Quantification

Fluorescence imaging was performed on a Zeiss AxioObserver Z.1 microscope, using a 20 × dry objective with 0.8 numerical aperture (NA) or 63 × oil objective with 1.4 NA, coupled to a Zeiss HRm AxioCam. In each repetition, cells were cultured and stained simultaneously and imaged using identical settings.

Fluorescence intensity was quantified using the Fiji image analysis software and the researcher was blind to the genotype. Sholl analysis was performed using the open-source SNT plugin of Fiji, and tracings of the dendrites were used to calculate TDL, number of primary dendrites, branching points and endpoints. MAP2 staining was used to measure the soma area. The distance between MAP2 staining and Ankyrin G (AnkG) staining was used to determine the distance of the AIS from the soma. The length of the AIS was determined by measuring the distance between the first and the last point along the axon in which AnkG staining decreases to 0.33 of the maximum fluorescence intensity, as described in Beros et al. (2024). Neurons with multiple AISs were excluded from the analysis.

Quantification of synaptic protein (PSD95, vesicular glutamate transporter type 1 (vGluT1) and surface glutamate receptors) immunoreactivity was performed as previously described (Afonso et al. 2019). Briefly, regions of interest (ROIs) were manually traced around isolated secondary dendrites. For each dendrite, we considered the longest segment possible. Ideally, this is equivalent to the segment from the branching point between the primary and secondary dendrite up to the first branching point with a tertiary dendrite. Portions of the dendrite that were close to bright aggregates of antibodies (due to the live staining protocol) were excluded, to avoid biases in the quantification. For this reason, in some cases, multiple ROIs were traced on the same secondary dendrite. In addition, we tried to include all the measurable dendrites for each cell. The protein of interest was analysed after setting a user-defined threshold; the recognisable puncta were counted, and the intensity was measured. To focus on glutamate receptor clusters located within presumably active synapses, PSD95 and vGluT1 signals were thresholded as well, and only the receptor clusters that colocalised simultaneously with PSD95 and vGluT1 were included in the analysis. Data were normalised on the length of the analysed dendritic segment, measured on the basis of MAP2 staining.

Fluorescence imaging was performed at the MICC Imaging facility of CNC-UC, *partially supported by PPBI—Portuguese Platform of BioImaging (PPBI-POCI-01–0145-FEDER-022122).*

### Multielectrode Array Recording and Analysis

Prior to start, multiwells were visually inspected to ensure that cultured neurons grew homogeneously on the array of electrodes and the cell density was comparable across genotypes. Spontaneous electrical activity of neurons was recorded using Maestro Edge system (Axion BioSystems) with AxIS Navigator software (Axion BioSystems). Recordings were carried out in NBM-A (GIBCO) supplemented with 0.12 mg/ml gentamicin (GIBCO), 2% B27 (GIBCO) and 0.5 mM l-glutamine. Neurons were allowed to stabilise in the system for 10 min prior to record for 5 min at 37 °C and 5% CO_2_ and 95% air. The AxIS Neural Metrics Tool Software (Axio BioSystems) was used to extract and analyse the traces. Coincidence artefacts were removed, and electrodes were considered active if detecting more than five spikes per minute. Spikes were defined as variations greater than 5.5 standard deviations of the noise. Bursts were defined as groups of at least five spikes with an inter-spike interval lower than 100 ms.

### Statistical Analysis

Prism 8 software (Graphpad) was used to visualise the data and to perform statistical analysis. Data were normalised to control conditions, and the Shapiro–Wilk test was used to test normality. If normality was confirmed, data were represented as mean ± standard error of the mean (SEM). Outliers were removed with the ROUT test (*Q* = 1%), and statistical analysis was performed using a two-tailed unpaired Student’s *t*-test. If criteria for normality were not met, the median and interquartile range were presented and a two-tailed unpaired Mann–Whitney test was used. To assess the correlation between different morphometric parameters, the nonparametric Spearman correlation coefficient was computed. Significance of each Spearman coefficient was tested by calculating the corresponding two-tailed *p* value.

## Results

### FMRP Regulates Neuronal Morphology in Cultured Neurons

**Fig. 1:**
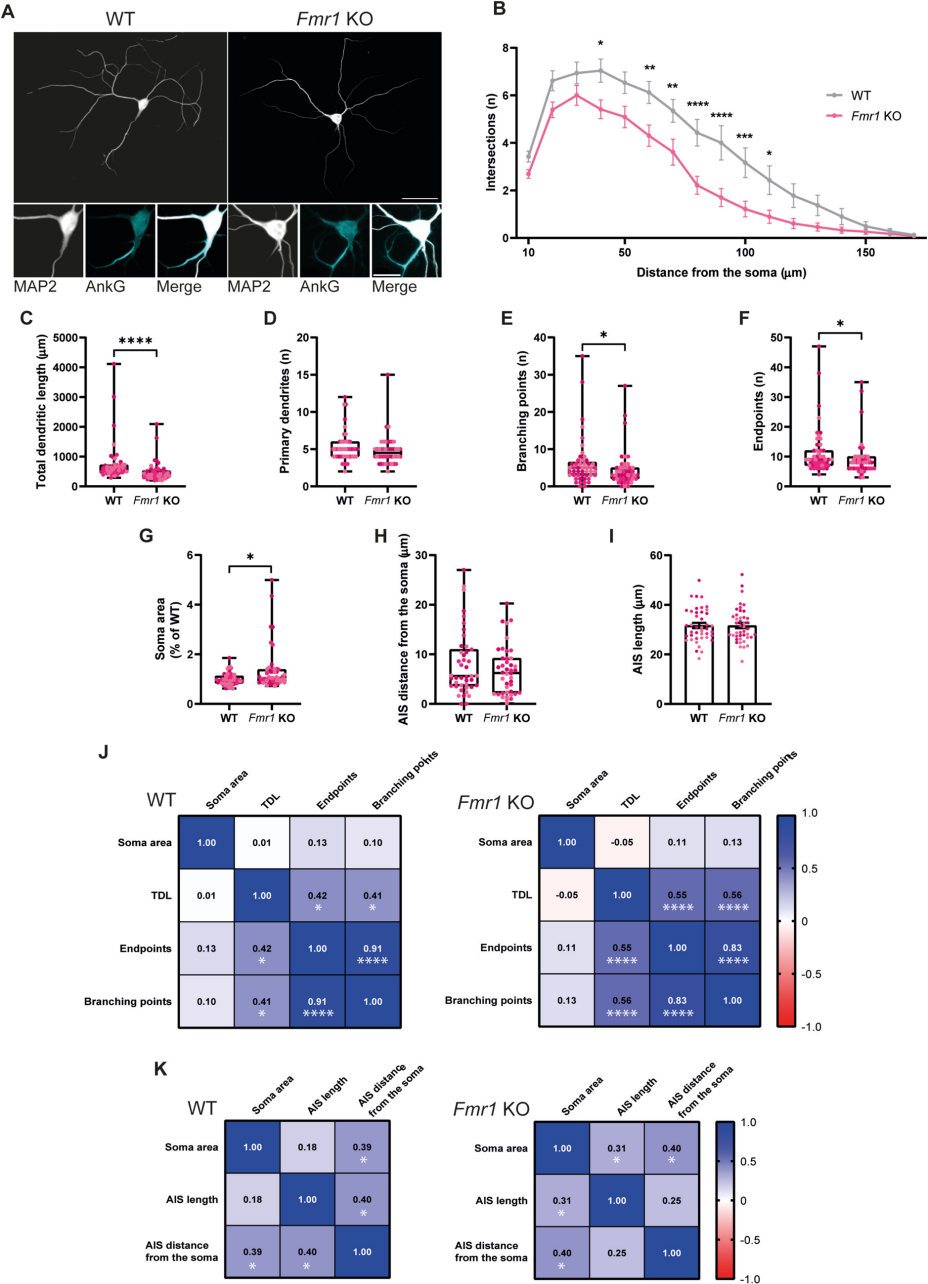
Morphological characterisation of cultured *Fmr1* KO hippocampal neurons. WT and *Fmr1* KO cultured hippocampal neurons (DIV15) immunolabelled for MAP2 (scale bar, 50 µm) (**A**). Insert: somata and axonal initial segments (AIS) of cultured WT and *Fmr1* KO hippocampal neurons, immunolabelled for MAP2 and AnkG, respectively (scale bar, 10 µm). Sholl analysis of cultured WT and *Fmr1* KO hippocampal neurons (**B**). Data are expressed as mean ± SEM and analysed with a mixed-effect analysis with Sidak correction (*p < 0.05; **p < 0.01; ***p < 0.001; ****p < 0.0001). Quantification of total dendritic length (TDL) (**C**), primary dendrites (**D**), branching points (**E**), endpoints (F) and soma area (**G**) of cultured WT and *Fmr1* KO hippocampal neurons. *n* = 49 WT and *n* = 46 *Fmr1* KO neurons were analysed, from *N* = 3 independent preparations. Quantification of the distance between the AIS and the soma (**H**) and the length of the AIS (**I**) in cultured WT and *Fmr1* KO hippocampal neurons. *n* = 42 WT and *n* = 43 *Fmr1* KO neurons were analysed, from *N* = 3 independent preparations. Each data point represents the results obtained in a neuron and each colour indicates a different preparation (**C**–**I**). Two-tailed Mann–Whitney test was used to compare WT and *Fmr1* KO conditions (*p < 0.05; ****p < 0.0001). Correlation matrix between the soma area, TDL, number of endpoints and branching points in cultured WT and *Fmr1* KO hippocampal neurons (**J**). Correlation matrix between the soma area, AIS length and AIS distance from the soma in cultured WT and *Fmr1* KO hippocampal neurons (**K**). For each correlation, the Spearman coefficient is reported, while the *p* value is reported only when significant (*p < 0.05; ****p < 0.0001)

To determine the effect of the lack of FMRP on the morphology of neurons differentiated in vitro, low-density primary hippocampal cultures were prepared from postnatal day 0–1 (P0-P1) WT and *Fmr1* KO male mice. On day in vitro (DIV) 15, neurons were immunolabelled for the cytoskeletal marker MAP2, to visualise the somatodendritic compartment, and for Ankirin G (AnkG) to identify the axonal initial segment (AIS) (Fig. 1A). To study the effects of the lack of FMRP on dendritic arborisation, we traced dendrites of WT and *Fmr1* KO neurons and performed Sholl analysis. The dendritic tree of *Fmr1* KO neurons displayed a decreased complexity compared to WT neurons, especially from 60 to 100 µm away from the soma (Fig. 1B); the total dendritic length (TDL) was also reduced in neurons lacking FMRP compared to WT neurons (Fig. 1C). Although no significant differences were detected in the number of primary dendrites (Fig. 1D), there was a reduction in the number of branching points (Fig. 1E) and endpoints (Fig. 1F) in *Fmr1* KO neurons. In addition, *Fmr1* KO neurons showed bigger somata than the WT control (Fig. 1G). Together, these data support the hypothesis that FMRP regulates the complexity of the dendritic tree and the soma size in differentiated hippocampal neurons. Finally, we focused on the AIS, namely the initial part of the axon in which voltage-gated sodium and potassium channels are clustered and action potentials are generated (Rapp et al. 1996; Stuart et al. 1997). We found no difference across genotypes in the distance between the AIS and the soma (Fig. 1H), as well as in the AIS length (Fig. 1I). These data suggest that FMRP is not involved in the regulation of AIS morphology.

Since *Fmr1* KO neurons display a larger soma, that acts as a capacitor accumulating more charge (Limon et al. 2005), but a less complex dendritic tree, that potentially allows for a minor dispersion of postsynaptic potentials (Magee 2000; Rinzel and Rall 1974), we asked whether there could be a compensatory mechanism to correct excitability. To address this question, we correlated the area of the soma with the total dendritic length, the number of endpoints and branching points in WT and *Fmr1* KO neurons, by calculating the Spearman coefficient (Fig. 1J). In WT neurons, we found a significant moderate positive correlation between the total dendritic length and the number of endpoints, as well as between the total dendritic length and the number of branching points. We also found a strong correlation between the number of endpoints and the number of branching points, as expected, but there was no correlation between the soma area and the total dendritic length, number of endpoints or number of branching points. In *Fmr1* KO neurons, we observed correlations similar to those in WT neurons: more specifically, a moderate positive correlation between total dendritic length and the number of endpoints, as well as between total dendritic length and the number of branching points. Moreover, we observed a positive correlation between the number of endpoints and branching points, while no correlation was found between the soma area and the total dendritic length, number of endpoints or number of branching points.

Since another key factor that controls neuronal excitability is the morphology of the AIS, and considering that the AIS shows great plasticity (Jenkins and Bender 2024; Yamada and Kuba 2016), we hypothesised that neurons could adjust the length and the distance from the soma of the AIS to finely tune excitability. To address this question, we correlated the soma area with the AIS length and distance from the soma in WT and *Fmr1* KO neurons (Fig. 1K), despite not having observed any difference across genotypes (Fig. 1H and I). In WT neurons, we found a significant positive correlation between the area of the soma and the distance of the AIS from the soma and between the AIS length and distance from the soma. Interestingly, in *Fmr1* KO neurons, there was a significant positive correlation between the soma area and the AIS distance from the soma, as well as between the soma area and the AIS length. On the other hand, *Fmr1* KO neurons did not show any correlation between AIS length and distance from the soma.

### Synaptic Density Is Not Affected in Mature Cultured Fmr1 KO Neurons

**Fig. 2:**
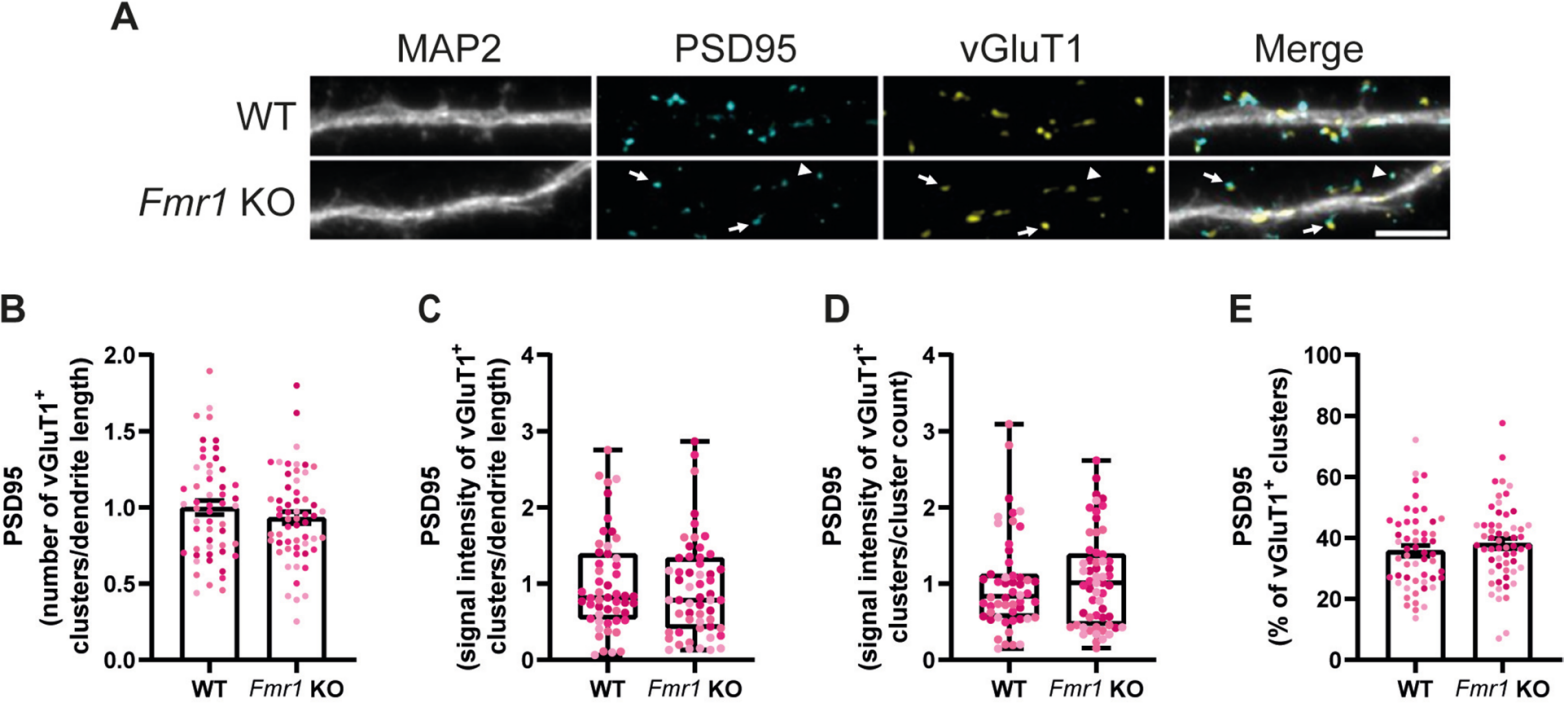
The distribution of active synapses is not affected in cultured *Fmr1* KO hippocampal neurons. Cultured WT and *Fmr1* KO hippocampal neurons (DIV15) immunolabelled for MAP2, PSD95 and vGluT1 (scale bar, 5 µm) (**A**). Arrows indicate vGluT1 positive-PSD95 clusters, while arrowheads indicate vGluT1 negative-PSD95 clusters. Quantification of the number of vGluT1 positive-PSD95 clusters per dendrite length (**B**), signal intensity of vGluT1 positive-PSD95 staining per dendrite length (**C**) and of the signal intensity per cluster (**D**). Percentage of the PSD95 clusters that colocalise with vGluT1 (**E**). *n* = 53 WT and *n* = 58 *Fmr1* KO neurons were analysed, from *N* = 4 independent preparations. Data are represented with a bar plot displaying mean ± SEM and were analysed with a two-tailed unpaired Student’s *t*-test, whenever the criteria for a normal distribution are met. In case of a non-normal distribution, the box and whiskers plot is used to represent the data, and a two-tailed Mann–Whitney test is used to compare WT and *Fmr1* KO conditions

Alterations in spine density and maturation are a major hallmark of FXS, but such alterations seem to be age- and brain-region dependent (extensively reviewed in He and Portera-Cailliau (2013)). Accordingly, FMRP regulates the mRNA of PSD95 and many proteins necessary for a proper synaptic stabilisation and function (Darnell et al. 2011). For this reason, we hypothesised that cultured *Fmr1* KO neurons could show differences in synaptic density and glutamate receptor organisation, when compared to WT neurons. To address this question, we immunolabelled WT and *Fmr1* KO hippocampal neurons with antibodies against MAP2, the postsynaptic marker PSD95 and the presynaptic marker vesicular glutamate transporter 1 (vGluT1). We measured the number (Fig. 2B) and signal intensity (Fig. 2C) of PSD95 clusters per dendritic length, as well as the signal intensity per cluster (Fig. 1D). We focused specifically on presumably active synapses, by limiting the analysis only to PSD95 clusters that colocalised with vGluT1 (Fig. 2A, arrows). No significant difference was detected across genotypes in any of the parameters quantified. Finally, WT and *Fmr1* KO neurons displayed a similar percentage of presumably active synapses (Fig. 2E), determined as the ratio of vGluT1^+^-PSD95 clusters to the total number of PSD95 clusters.

Quantification of the total number of PSD95 clusters and of the total PSD95 signal intensity per dendrite length (Fig. 2A, arrows and arrowheads, supplementary Fig. S1A and B), and of the immunoreactivity per PSD95 cluster (Supplementary Fig. S1C), did not show statistically significant differences between WT and *Fmr1* KO neurons. We also quantified the same parameters for vGluT1 (Supplementary Fig. S1D-F), and we did not observe any differences between WT and *Fmr1* KO neurons. Taking together these pieces of evidence, we can conclude that synaptic density is not affected in cultured *Fmr1* KO neurons.

### FMRP Specifically Regulates the Synaptic Expression of GluA2-AMPA Receptor Subunits

**Fig. 3:**
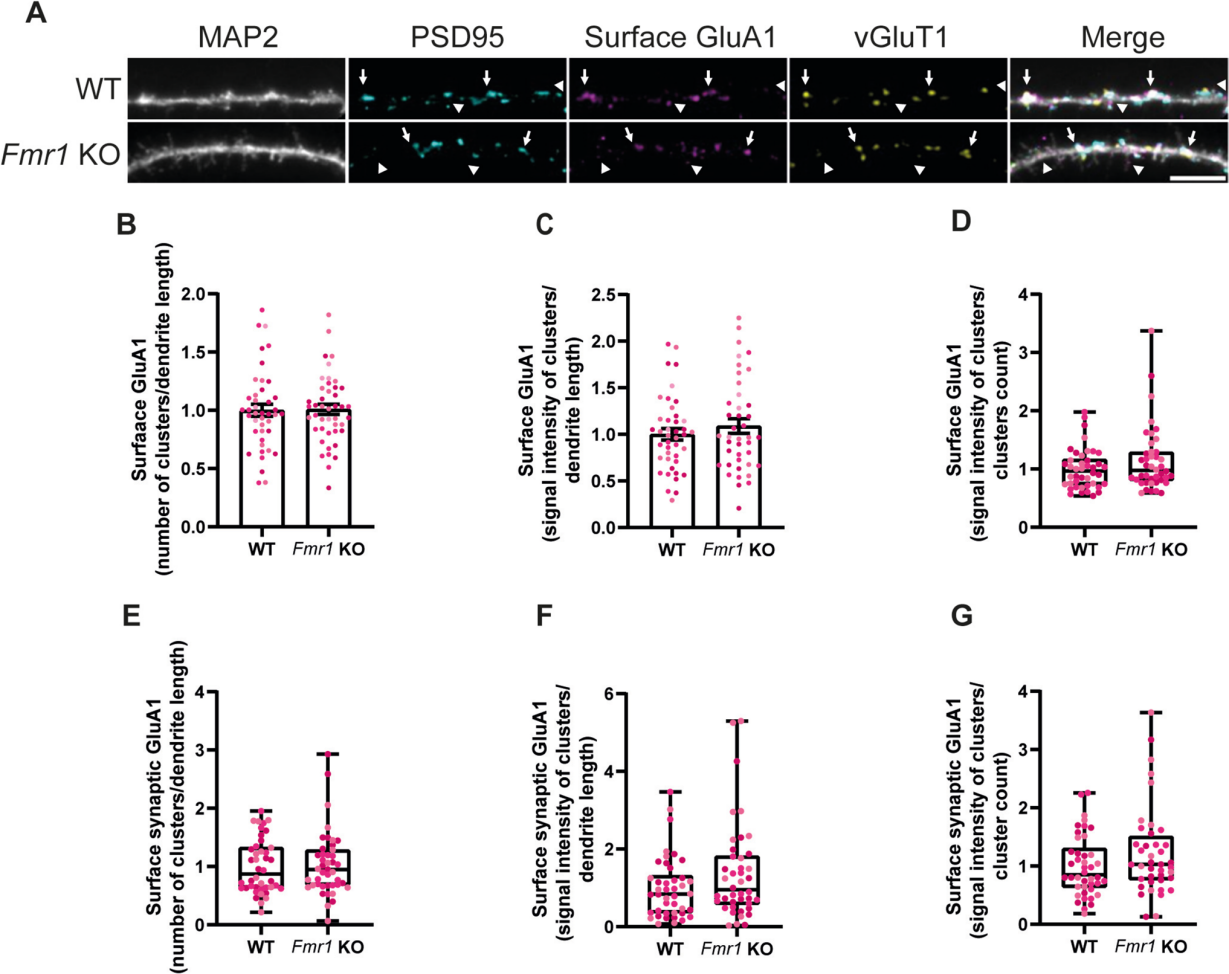
GluA1 distribution on the neuronal surface is not affected in cultured *Fmr1* KO hippocampal neurons. Representative images of DIV15 WT and *Fmr1* KO hippocampal neurons immunolabelled for MAP2, PSD95, surface GluA1 and vGluT1 (scale bar, 5 µm) (**A**). Arrows indicate synaptic GluA1, while arrowheads indicate extrasynaptic GluA1. Quantification of the number of surface GluA1 clusters per dendrite length (**B**), signal intensity of surface GluA1 staining per dendrite length (**C**) and signal intensity per cluster (**D**). Quantification of the number of synaptic surface GluA1 clusters per dendrite length (**E**), signal intensity of synaptic surface GluA1 staining per dendrite length (**F**) and signal intensity per synaptic GluA1 cluster (**G**). Synaptic localisation was assessed by simultaneous colocalisation with PSD95 and vGluT1. *n* = 42 WT and *n* = 44 *Fmr1* KO neurons were analysed, from *N* = 3 independent preparations. Each data point represents a neuron, and each colour indicates a different preparation. Data are expressed as mean ± SEM and were analysed with a two-tailed unpaired Student’s *t*-test, whenever the criteria for a normal distribution were met (**B**–**G**). In case of a non-normal distribution, a two-tailed Mann–Whitney test was used to compare WT and *Fmr1* KO conditions

AMPA receptors are widely expressed in the brain (Martin et al. 1993) and are formed by the heteromerisation of four subunits, GluA1 to GluA4 (Corti and Duarte 2023), but GluA1 and GluA2 are the most abundant subunits in hippocampal neurons (Wenthold et al. 1996). AMPA receptor abundancy in the synaptic plasma membrane is tightly regulated, being a major determinant of synaptic strength and spine architecture (Gomes et al. 2003; McKinney et al. 1999). In order to elucidate the role of FMRP in regulating GluA1 distribution on the neuronal surface under resting conditions, WT and *Fmr1* KO neurons (DIV15) were incubated with a primary antibody directed against an extracellular epitope of GluA1 under non-permeabilising conditions (Fig. 3A, arrows and arrowheads). No differences were found between WT and *Fmr1* KO neurons when analysing the number (Fig. 3B) or signal intensity (Fig. 3C) of total surface GluA1 clusters per dendrite length, as well as the signal intensity per cluster (Fig. 3D). Then, we hypothesised that FMRP-deficient neurons could exhibit a dysregulation in surface GluA1 distribution selectively at the synapses. To answer this question, the analysis was restricted to GluA1 clusters that simultaneously colocalised with the synaptic markers PSD95 and vGluT1 (Fig. 3A, arrows). We found that WT and *Fmr1* KO neurons did not show significant differences in terms of number (Fig. 3E) and signal intensity (Fig. 3F) of surface synaptic GluA1 clusters per dendrite length. The signal intensity of synaptic clusters per cluster count was comparable across genotypes (Fig. 3G). Together, these data indicate that FMRP is not involved in the regulation of the distribution of GluA1 in the neuronal surface in cultured hippocampal neurons.

**Fig. 4:**
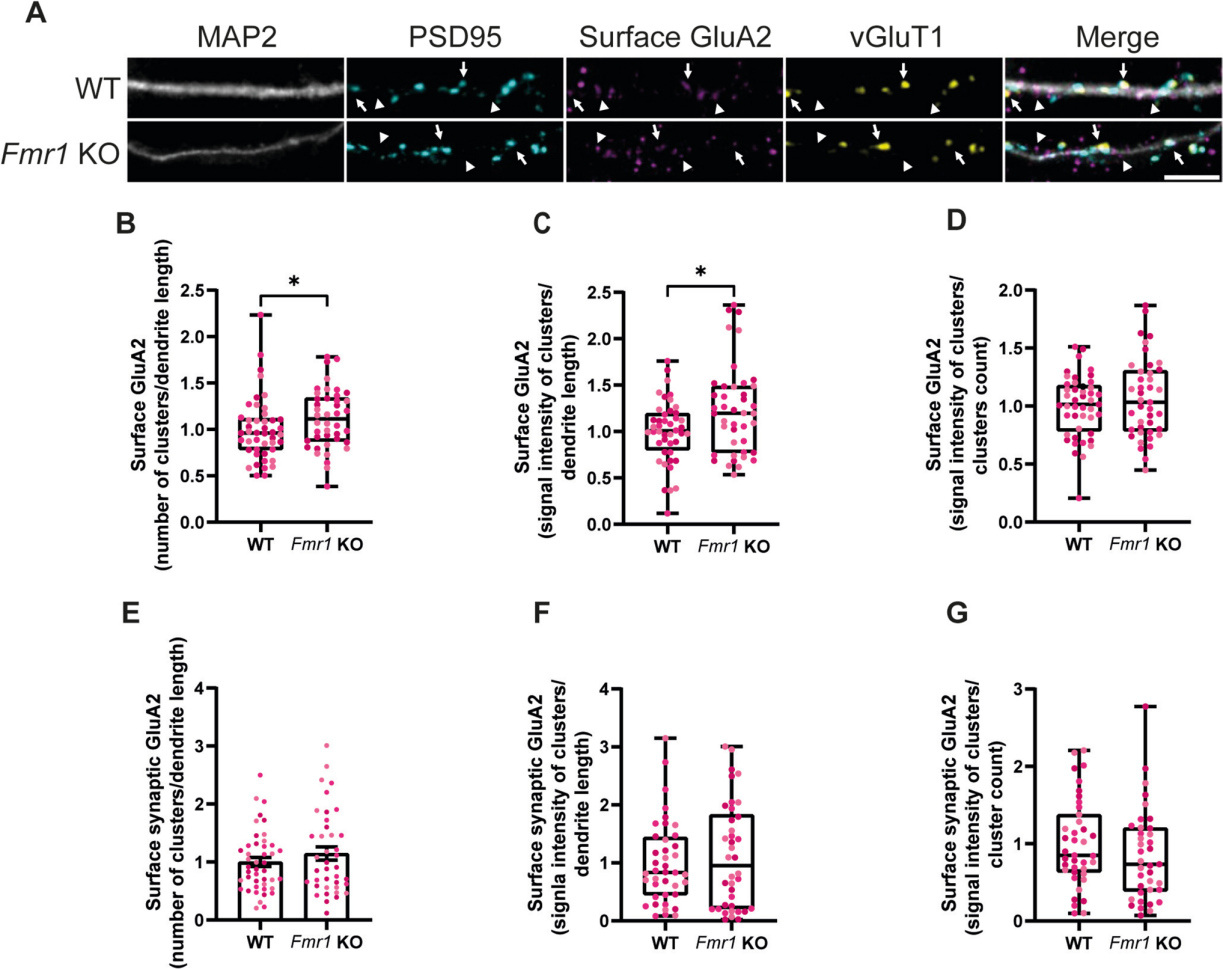
GluA2 is more abundant on the surface of cultured *Fmr1* KO hippocampal neurons, but the synaptic content is not altered. Representative images of WT and *Fmr1* KO hippocampal neurons (DIV15) immunolabelled for MAP2, PSD95, surface GluA2 and vGluT1 (scale bar, 5 µm) (**A**). Arrows indicate synaptic GluA2, while arrowheads indicate extrasynaptic GluA2. Quantification of the number of surface GluA2 clusters per dendrite length (**B**), signal intensity of surface GluA2 staining per dendrite length (**C**) and signal intensity per cluster (**D**). Quantification of the number of synaptic surface GluA2 clusters per dendrite length (**E**), signal intensity of synaptic surface GluA2 staining per dendrite length (**F**) and signal intensity per synaptic GluA2 cluster (**G**). Synaptic localisation was assessed by simultaneous colocalisation with PSD95 and vGluT1. *n* = 45 WT and *n* = 42 *Fmr1* KO neurons were analysed, from *N* = 3 independent preparations. Each data point represents a neuron and each colour indicates a different preparation. A two-tailed Mann–Whitney test was used to compare WT and *Fmr1* KO conditions in case of a non-normal distribution (*p < 0.05) (**B**–**D**, **F**, **G**). Data were expressed as mean ± SEM and analysed with a two-tailed Student’s unpaired *t*-test (**E**)

GluA2 is the only subunit of AMPA receptors that is not permeable to calcium, making it a very important player in the control of many Ca^2+^-dependent processes (Man 2011). We investigated the GluA2 surface expression in cultured WT and *Fmr1* KO hippocampal neurons (DIV15) by incubating the cells with a primary antibody directed against an extracellular epitope of the AMPA receptor subunit under non-permeabilising conditions (Fig. 4A, arrows and arrowheads). We observed that *Fmr1* KO neurons exhibit a higher number of GluA2 clusters (Fig. 4B) and increased GluA2 content in the plasma membrane (Fig. 4C), but the amount of protein per cluster was comparable to WT neurons (Fig. 4D). We then investigated whether this generalised increase in surface GluA2 affects the synaptic content of this AMPA receptor subunit. To address this question, we restricted our analysis on GluA2 clusters that simultaneously colocalised with the synaptic markers PSD95 and vGluT1 (Fig. 4A, arrows). Interestingly, we found that *Fmr1* KO neurons displayed the same number (Fig. 4E) and signal intensity (Fig. 4F) of synaptic surface GluA2 clusters, as well as the same signal intensity per cluster (Fig. 4G), when compared to WT neurons. Taken together, these data show a role for FMRP in regulating the surface content, but not the synaptic content, of GluA2.

### NMDA Receptor Distribution Is Impaired in Fmr1 KO Cultured Hippocampal Neurons

NMDA receptors for glutamate are fundamental for a proper synaptic development and plasticity (Barkus et al. 2012). These receptors are tetrameric ion channels composed by two obligatory GluN1 subunits and two GluN2 or GluN3 subunits (Hansen et al. 2021). In the hippocampus, GluN2B is widely expressed during development and partially substituted by GluN2A as neurons mature (Wenzel et al. 1997). Notably, FMRP binds the mRNAs of both GluN2A and GluN2B (Darnell et al. 2011).

**Fig. 5:**
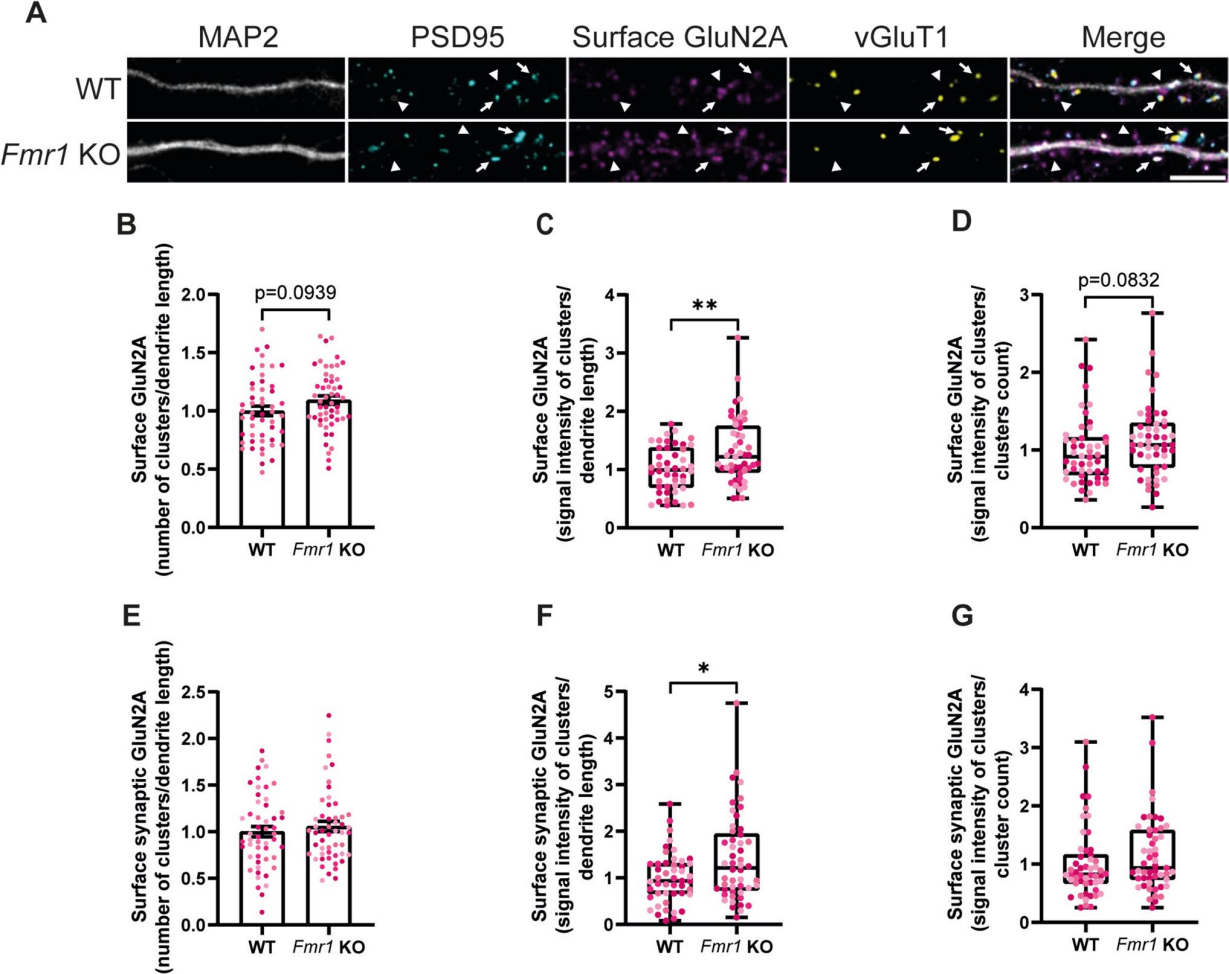
GluN2A is more abundant on the synaptic surface of cultured *Fmr1* KO hippocampal neurons. Representative images of cultured WT and *Fmr1* KO hippocampal neurons (DIV15) immunolabelled for MAP2, PSD95, surface GluN2A and vGluT1 (scale bar, 5 µm) (**A**). Arrows indicate synaptic GluN2A, while arrowheads indicate extrasynaptic GluN2A. Quantification of the number of surface GluN2A clusters per dendrite length (**B**), signal intensity of surface GluN2A staining per dendrite length (**C**) and signal intensity per cluster (**D**). Quantification of the number of synaptic surface GluN2A clusters per dendrite length (**E**), signal intensity of synaptic surface GluN2A staining per dendrite length (**F**) and signal intensity per synaptic surface GluN2A cluster (**G**). Synaptic localisation was assessed by simultaneous colocalisation with PSD95 and vGluT1. *n* = 51 WT and *n* = 52 *Fmr1* KO neurons were analysed, from *N* = 4 independent preparations. Each data point represents a neuron and each colour indicates a different preparation. Data are expressed as mean ± SEM and analysed with a two-tailed unpaired Student’s *t*-test, whenever the criteria for a normal distribution were met (**B**–**G**). In case of a non-normal distribution, a two-tailed Mann–Whitney test was used to compare WT and *Fmr1* KO conditions

To understand if FMRP contributes to regulate the surface distribution of GluN2A, we incubated live WT and *Fmr1* KO neurons with an antibody that recognises an extracellular epitope in GluN2A N-terminus under non-permeabilising conditions (Fig. 5A, arrows and arrowheads). We then measured the number and signal intensity of GluN2A clusters per dendritic length and the signal intensity per cluster. We observed a slight increase in the number of surface GluN2A clusters in *Fmr1* KO hippocampal neurons, but the difference was not statistically significant (Fig. 5B). Nonetheless, we detected an upregulation in total surface GluN2A signal intensity in neurons lacking FMRP (Fig. 5C), indicating that more GluN2A is found in the neuronal plasma membrane under these conditions. At the same time, there was a slight increase in the signal intensity of each cluster (Fig. 5D), but the difference did not reach statistical significance. We then asked if this increase in surface GluN2A could impact the synaptic content of this receptor in hippocampal neurons lacking FMRP. This was assessed by analysing the GluN2A clusters that colocalised simultaneously with PSD95 and vGluT1 (Fig. 5A, arrows). The results showed a similar number of synaptic surface GluN2A clusters in WT and *Fmr1* KO neurons (Fig. 5E). Moreover, surface GluN2A signal intensity per dendrite length was increased in *Fmr1* KO neurons (Fig. 5F), but this increase was not sufficient to significantly affect the signal intensity per cluster (Fig. 5G). Taken together, these results show that FMRP regulates the abundance of GluN2A in the neuronal plasma membrane and in the synaptic compartment in cultured hippocampal neurons.

**Fig. 6:**
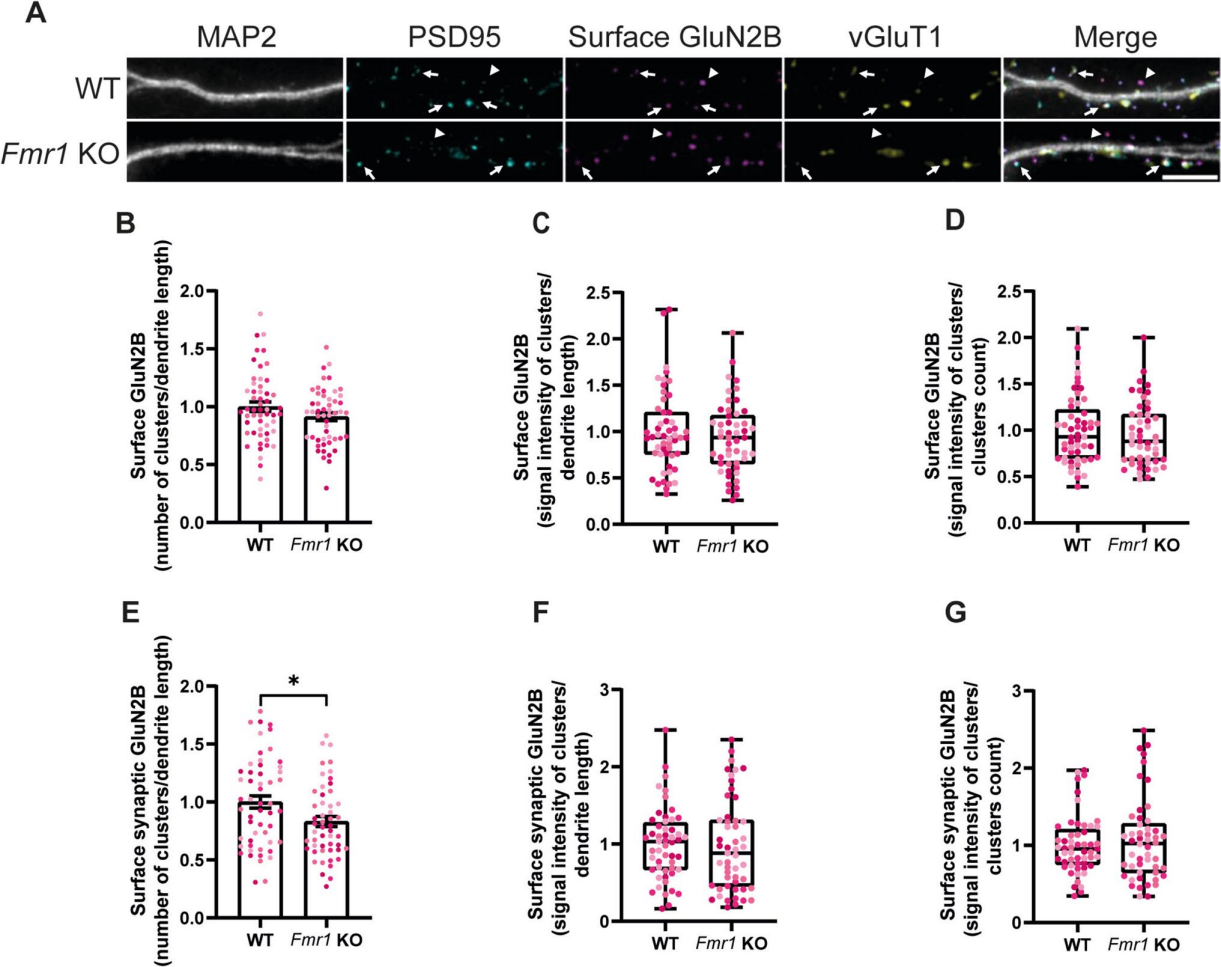
GluN2B is less abundant on the synaptic surface of *Fmr1* KO primary hippocampal neurons. Representative images of cultured WT and *Fmr1* KO hippocampal neurons (DIV15) immunolabelled for MAP2, PSD95, surface GluN2B and vGluT1 (scale bar, 5 µm) (**A**). Arrows indicate synaptic GluN2B, while arrowheads indicate extrasynaptic GluN2B. Quantification of the number of surface GluN2B clusters per dendrite length (**B**), signal intensity of surface GluN2B staining per dendrite length (**C**) and signal intensity per cluster (**D**). Quantification of the number of synaptic surface GluN2B clusters per dendrite length (**E**), signal intensity of synaptic surface GluN2B staining per dendrite length (**F**) and signal intensity per synaptic surface GluN2B cluster (**G**). Synaptic localisation was assessed by simultaneous colocalisation with PSD95 and vGluT1. *n* = 53 WT and *n* = 50 *Fmr1* KO neurons were analysed, from *N* = 4 independent preparations. Each data point represents a neuron and each colour indicates a different preparation. Data are expressed as mean ± SEM and analysed with a two-tailed unpaired Student’s *t*-test, whenever the criteria for a normal distribution were met (**B**–**G**). In case of a non-normal distribution, a two-tailed Mann–Whitney test was used to compare WT and *Fmr1* KO conditions

In complementary experiments, we analysed the distribution of GluN2B subunits of NMDAR receptors (Fig. 6A, arrows and arrowheads), and we found no differences across genotypes in the number (Fig. 6B) and signal intensity (Fig. 6C) per dendrite length of surface GluN2B clusters. In addition, no differences were found in GluN2B signal intensity per cluster (Fig. 6D). Surprisingly, when considering only the synaptic fraction of GluN2B clusters, we observed that *Fmr1* KO neurons had a lower number of GluN2B clusters in the synapses (Fig. 6E, arrows), but the signal intensity per dendrite length (Fig. 6F) and the signal intensity per cluster (Fig. 6G) were comparable to control. These pieces of evidence point towards a role for FMRP in regulating the distribution of GluN2B-containing NMDA receptors within synapses.

### Spontaneous Firing Activity Is Decreased in Fmr1 KO Hippocampal Neurons

**Fig. 7:**
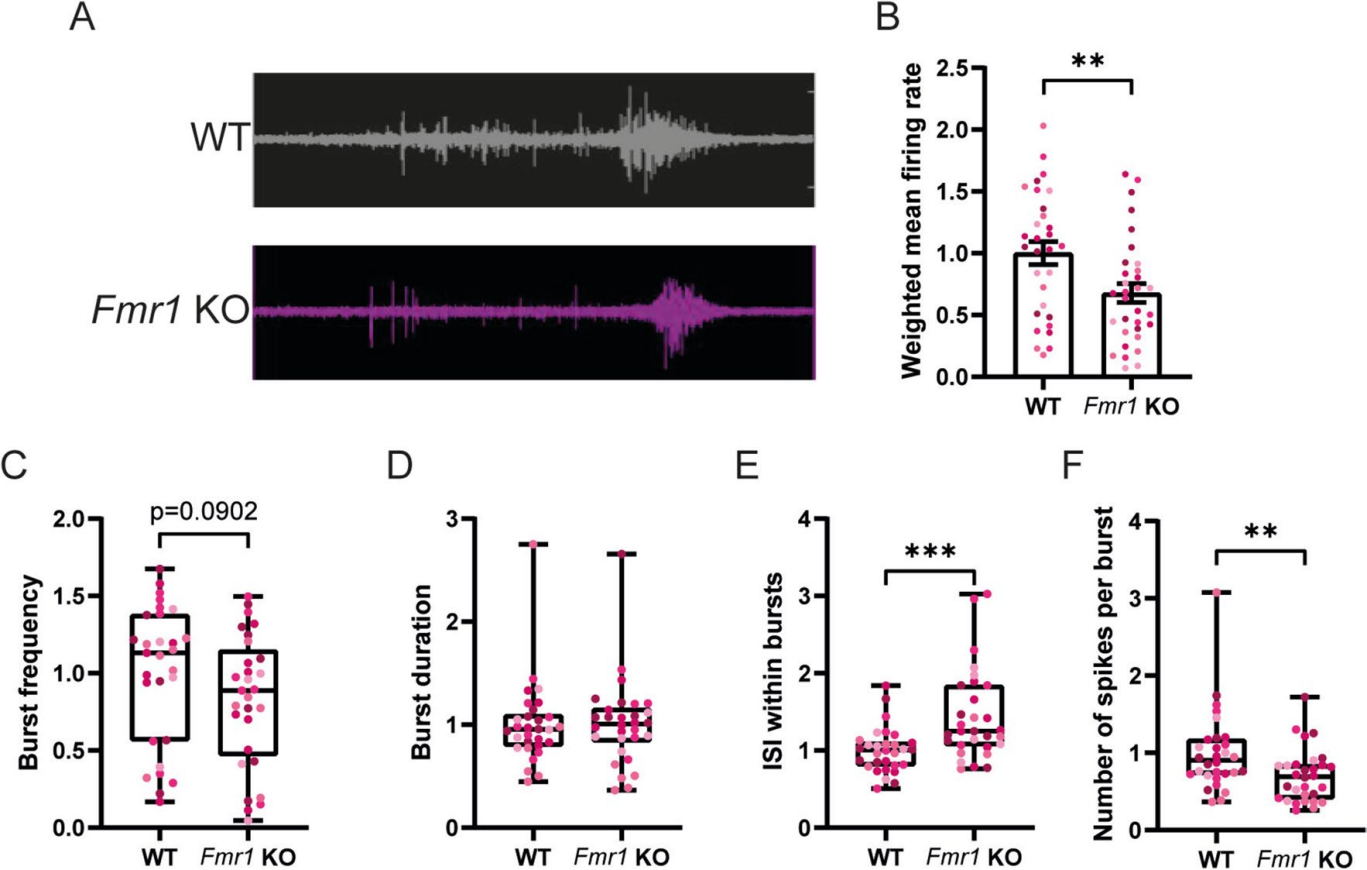
*Fmr1* KO hippocampal neurons display altered firing properties. **A** Representative traces of spontaneous activity of cultured WT and *Fmr1* KO primary hippocampal neurons (DIV15). **B** Quantification of the mean firing rate weighted on the number of active electrodes. **C** Quantification of the burst frequency, **D** burst duration, **E** inter-spike interval (ISI) within bursts and **F** number of spikes per burst. *n* = 30 wells with WT neurons and *n* = 30 wells with *Fmr1* KO neurons were analysed, from *N* = 5 independent preparations (6 wells per genotype per experiment). Each data point represents a well, and each colour indicates a different preparation. Data are expressed as mean ± SEM and analysed with a two-tailed unpaired Student’s *t*-test, whenever the criteria for a normal distribution were met (**B**–**E**). In case of a non-normal distribution, a two-tailed Mann–Whitney test was used to compare WT and *Fmr1* KO conditions

To assess how the differences in passive and active properties of *Fmr1* KO neurons impact neuronal firing, we recorded spontaneous neuronal activity with microelectrode arrays (MEAs) (Fig. 7A). We found an overall reduction in the mean firing rate corrected for the number of active electrodes in *Fmr1* KO neurons (Fig. 7B). Then, we evaluated the capability of FMRP-deficient neurons to produce firing patterns by looking at bursts. On one hand, *Fmr1* KO neurons exhibited burst frequency (Fig. 7C) and duration (Fig. 7D) comparable to control neurons. However, FMRP deficiency increased the inter-spike interval (ISI) within bursts (Fig. 7E) and reduced the number of spikes per burst (Fig. 7F). Taken together, these results show that *Fmr1* KO hippocampal neurons present functional alterations in their firing properties and in the capability to fire patterns of action potentials.

## Discussion

Although FXS is known to arise from the deficiency of FMRP, the functional consequences of the lack of this RNA-binding protein on brain physiology are not fully elucidated. To investigate this problem, we used primary cultures of hippocampal neurons from WT and *Fmr1* KO mice, to obtain low-density cultures composed mainly by pyramidal neurons, with a limited number of inhibitory neurons and no microglia (Kaech and Banker 2006). This method allowed to selectively address cell-autonomous consequences of FMRP deficiency on neuronal morphology, synaptic density and surface expression of glutamate receptors. We found that neurons lacking FMRP were characterised by a shorter and less complex dendritic tree and increased soma size, while the length of the axonal initial segment (AIS) and its distance from the soma were comparable to control neurons. Despite this, we found that the soma size and the positioning of the AIS positively correlated, but only in *Fmr1* KO neurons. Moreover, the density of excitatory synapses and the expression of GluA1 and GluA2 in the postsynaptic membrane were unchanged upon *Fmr1* KO, but neurons lacking FMRP presented more GluN2A and less GluN2B in the postsynaptic membrane. Finally, we observed a reduction of spontaneous activity in *Fmr1* KO neurons compared to control.

The observed decrease in dendritic length in cultured *Fmr1* KO hippocampal neurons at DIV15 is in accordance with what has been reported for cultured DIV7 and DIV20 *Fmr1* KO neurons (Braun and Segal 2000), showing stable alterations throughout neuronal development. *Fmr1* KO neurons had a lower number of branching points and endpoints but not of primary dendrites. Thus, the lack of FMRP negatively affects higher-order dendrites in a selective manner. Interestingly, *MeCP2* KO neurons, a model of the neurodevelopmental disorder Rett syndrome, display a reduction of the number of primary dendrites at DIV6 or DIV9, without significant alteration in the number of secondary dendrites. Such phenotype is switched in older neurons (DIV12 and DIV15) that present no alteration in the number of primary dendrites but a lower number of secondary dendrites (Baj et al. 2014). Moreover, the soma of *Fmr1* KO neurons was bigger compared to control neurons. Phosphatase and tensin homologue deleted on chromosome 10 (PTEN) is a putative mediator of the increase in soma volume observed in *Fmr1* KO neurons. Indeed, previous studies reported that PTEN regulates soma size via inhibiting the Akt/mTOR pathway in hippocampal granule neurons (Tariq et al. 2022). Interestingly, the mRNAs of both PTEN and mTOR are a target of FMRP (Darnell et al. 2011), and hyperactivity of the mTOR complex has been found in juvenile *Fmr1* KO mice (Sharma et al. 2010), as well as in FXS patients (Hoeffer et al. 2012). Furthermore, PTEN protein expression is increased in *Fmr1* KO hippocampi. This increase in PTEN directly contributes to the pathophysiology of FXS, since genetic deletion of PTEN restores the aberrant dendritic arborisation and spine density and length of *Fmr1* KO granule neurons (Sathyanarayana et al. 2022). In addition, mutations in the *PTEN* gene result in elevated expression and phosphorylation of FMRP in the hippocampus (Lugo et al. 2013) and were associated with autism spectrum disorder (ASD) (Conti et al. 2012).

Surprisingly, no differences were observed across genotypes regarding the positioning and length of the AIS, despite the fact that the main organiser of this region, Ankirin G (Bennett and Lambert 1999), is transcriptionally regulated by FMRP (Darnell et al. 2011). Considering the fact that larger neurons present higher membrane capacitance, while shorter dendrites allow an easier flow of current (Chavlis and Poirazi 2022), we hypothesised that the shortening of the dendritic tree observed in neurons lacking FMRP could be a compensatory mechanism that would allow counteracting the increase in soma size. However, to our surprise, we did not find any correlation between the morphometric parameters related to the dendritic tree and the soma. Instead, we observed a significant moderate positive correlation between the soma area and the AIS length in *Fmr1* KO neurons, but not in WT neurons. These alterations may allow adjusting neuronal excitability in neurons lacking FMRP. This view is supported by the available evidence showing that longer AIS contain more Na_v_1.2 channels (Freal et al. 2023). Moreover, an increase in persistent sodium current in the entorhinal cortex of *Fmr1* KO mice (Deng and Klyachko 2016) and an increase in Na^+^ conductance in L2/3 of the prefrontal cortex of *Fmr1* KO mice (Routh et al. 2017) have been reported. Finally, in this work, we observed a significant correlation between the AIS length and distance from the soma in WT neurons but not in *Fmr1* KO neurons. The AIS shows great plasticity in response to differences in neuronal morphology: in neurons harbouring a large dendritic tree, such as pyramidal neurons, excitability is enhanced when the AIS is longer and/or located distantly from the soma (Gulledge and Bravo 2016). The fact that this correlation is not present in *Fmr1* KO neurons points out that plasticity mechanisms that control excitability may be dysfunctional in FMRP-deficient neurons.

In this work, we also analysed the alterations in the density of active synapses in *Fmr1* KO neurons and characterised the surface expression of various AMPAR and NMDAR subunits. First, we found no difference in the density of active excitatory synapses between WT and *Fmr1* KO neurons, as determined by the colocalisation of vGluT1 and PSD95. This may be a consequence of the model used in this study: WT and *Fmr1* KO neurons were cultured on a feeder layer of WT astrocytes, which allow keeping a low density of neurons, adequate to study isolated dendrites. It was previously reported that the dendritic arbour area and the density of PSD95 and synaptophysin clusters are more developed in *Fmr1* KO neurons co-cultured with WT glia for 7 days compared to *Fmr1* KO neurons co-cultured with *Fmr1* KO glia (Jacobs and Doering 2010). The effects of a co-culture with WT astrocytes on the morphology of older *Fmr1* KO neurons (e.g. DIV15) are unknown, but we cannot exclude the presence of a trophic effect of WT glia on the synaptic density in *Fmr1* KO neurons measured in this work. We chose not to culture *Fmr1* KO neurons on a feeder layer of *Fmr1* KO astrocytes because it has been reported that co-culturing WT neurons with *Fmr1* KO astrocytes reduces the area of the dendritic arborisation and increases the number of active synapses at DIV7 and DIV14, when compared to WT neurons co-cultured with WT astrocytes (Jacobs et al. 2010). The latter effect of *Fmr1* KO astrocytes on synaptic development may account for our results showing no difference in the density of active excitatory synapses between WT and *Fmr1* KO neurons. Therefore, using genotype-matched astrocytes would not enable to selectively focus on the cell-autonomous effects of FMRP deletion, as it would introduce an additional variable, i.e. the astrocyte genotype. In addition to the effects described above, *Fmr1* KO astrocytes also decreased the rate of survival of co-cultured *Fmr1* KO neurons (Jacobs and Doering 2010).

Secondly, *Fmr1* KO neurons showed an upregulation in the total surface expression of GluA2, while the pattern of GluA1 distribution was not altered by the lack of FMRP. When focusing specifically on the synaptic compartment, we found that the distribution of AMPAR subunits in *Fmr1* KO neurons did not significantly differ from the distribution of AMPAR subunits in WT neurons. This contrasts with the results obtained in a previous study showing that the synaptic delivery of GluA1 is impaired in organotypic cortical slices prepared from P7 *Fmr1* KO mice, with a consequent reduction in LTP (Hu et al. 2008). In addition, silencing *Fmr1* expression with a siRNA increases GluA1 internalisation in cultured hippocampal neurons (Nakamoto et al. 2007), despite the fact that GluA1 mRNA is not among FMRP targets (Darnell et al. 2011). The effect of FMRP on the surface distribution of GluA1 may not be cell-autonomous given the results obtained in the present study showing no alterations in synaptic GluA1 surface expression in cultured *Fmr1* KO neurons. The similar GluA2 surface expression observed in the present work when cultured WT and *Fmr1* KO neurons were compared is in accordance with the results obtained in hippocampal synaptoneurosomes (subcellular fractions containing the pre- and postsynaptic regions) isolated from *Fmr1* KO mice (Chojnacka et al. 2023). However, they contrast with the evidence from previous studies reporting that synaptic GluA2 is upregulated in CA1 pyramidal neurons in organotypic slices prepared from P9 *Fmr1* KO mice, but such phenotype is reverted in organotypic slices prepared from P14 *Fmr1* KO mice (Banke and Barria 2020). Moreover, P21 *Fmr1* KO mice exhibit a higher expression of GluA2 in CA1 pyramidal neurons (Hwang et al. 2022), but it is unclear whether this oscillatory expression of GluA2 in FMRP-deficient neurons impacts the plasma membrane and synaptic expression of this subunit. This discrepancy may be due to different developmental stages of the animals used, but it may also hint that intracellular mechanisms responsible for the surface expression of GluA2 are distinct and at least partially independent from the ones regulating actual GluA2 synaptic content, for example through post-translational modifications (reviewed in Corti and Duarte (2023)). Since the results obtained in the present work showed comparable synaptic contents in GluA1 and GluA2 across genotypes, any defect in synaptic transmission and excitability observed in cultured *Fmr1* KO neurons is unlikely to result from AMPAR dysfunction. Whether the upregulation in total surface expression of GluA2 observed in dendrites of *Fmr1* KO neurons is relevant in the context of synaptic plasticity remains to be elucidated.

In the present work, we also found that the total surface expression of GluN2A, but not GluN2B, was upregulated in cultured *Fmr1* KO hippocampal neurons. In addition, synapses in cultured *Fmr1* KO neurons also presented a higher content of GluN2A, but the content of GluN2B was downregulated. Previous studies reported differences in the decay time of NMDAR-mediated excitatory postsynaptic currents in hippocampal slices from juvenile *Fmr1* KO mice, consistent with an early expression and insertion of GluN2A in hippocampal synapses during the development of *Fmr1* KO mice (Banke et al. 2024). Moreover, upregulation in the expression of GluN2A and GluN2B in total hippocampal lysates (Toft et al. 2016) and in the synaptosomal fraction (Lundbye et al. 2018) of *Fmr1* KO mice was reported, in accordance with the fact that FMRP directly regulates the protein synthesis of these two subunits (Darnell et al. 2011). Although an upregulation in GluN2A expression means a higher surface and synaptic content in *Fmr1* KO neurons, the same cannot be concluded for GluN2B in our experimental setting. This imbalance in NMDA receptor subunits may have deep consequences on dendritic processing of incoming inputs: in fact, GluN2A-containing NMDAR exhibit faster activation and deactivation kinetics and allow less charge transfer than GluN2B-containing NMDAR, making NMDAR-mediated excitatory postsynaptic currents smaller and faster when GluN2A is abundant (Vicini et al. 1998). It has actually been shown that *Fmr1* KO hippocampal slices present a weaker summation of stimuli delivered in close temporal proximity compared to control slices, e.g. in response to a paired-pulse stimulation (Banke et al. 2024). Moreover, NMDAR-mediated calcium entry is fundamental for the expression of plasticity through activation of CamKII (reviewed in Nicoll and Schulman (2023)). On one hand, GluN2A confers reduced calcium permeability to NMDAR when compared to GluN2B (Sobczyk et al. 2005), and the marked abundance of GluN2A in *Fmr1* KO neurons may explain the impairment in the expression of plasticity observed in FMRP-lacking animals (Lauterborn et al. 2007). Indeed, dampening the increased levels of GluN2A in *Fmr1* KO juvenile mice either by pharmacological inhibition or by crossing *Fmr1* KO mice with *Grin2A* KO mice restores both long-term potentiation (LTP) and long-term depression (LTD) mediated by metabotropic glutamate receptors mGluR5 (Lundbye et al. 2018). On the other hand, CamKII interaction with GluN2B is important in maintaining synaptic strength and expressing LTP (Sanhueza et al. 2011), and the decrease in GluN2B here reported in *Fmr1* KO synapses coupled with an hypoactivation of CamKII observed in *Fmr1* KO neurons (Chen et al. 2023) provides a further mechanism to explain deficits in the expression of plasticity in FMRP-deficient animals.

Lastly, our results also showed a reduction in spontaneous activity in cultures of *Fmr1* KO hippocampal neurons when compared to control neurons, as determined with MEA. In particular, we observed a reduction of the mean firing rate and the number of spikes per burst, while the burst duration and frequency were comparable to those observed in control neurons. This result may be a direct consequence of the lack of FMRP in cultured hippocampal neurons, coupled to the upregulation in synaptic surface expression of GluN2A-containing NMDAR, an increase in the soma size and/or a dysfunctional positioning of the AIS in respect to the soma size. These alterations are expected to make an effective depolarisation of the somatodendritic compartment more difficult to achieve, despite the fact that the dendritic tree is less complex and that AMPAR transmission was not changed in *Fmr1* KO hippocampal neurons. The effective contribution of these features in tuning input integration and neuronal excitability still remains to be determined and more studies are needed. Moreover, additional studies are required to determine how the cell-autonomous features in *Fmr1* KO hippocampal neurons are integrated within the more complex reality of a brain circuit, and how other cell types such as glial cells or interneurons turn this hypoexcitability of pyramidal neurons into hyperexcitability of neuronal circuits.

## Supplementary Information

Below is the link to the electronic supplementary material.

Supplementary Figure S1 (TIF 10281 KB)

Supplementary Table 1 (DOCX 18 KB)

## Data Availability

No datasets were generated or analysed during the current study.
