# Supplementary figures and images for: FMRP Controls Neuronal Architecture and Synaptic Content of NMDA Receptors in Cultured Hippocampal Neurons
Source: J Mol Neurosci. 2025 Apr 2;75(2):44. doi: 10.1007/s12031-025-02325-8 (PMC11965214; doi:10.1007/s12031-025-02325-8)

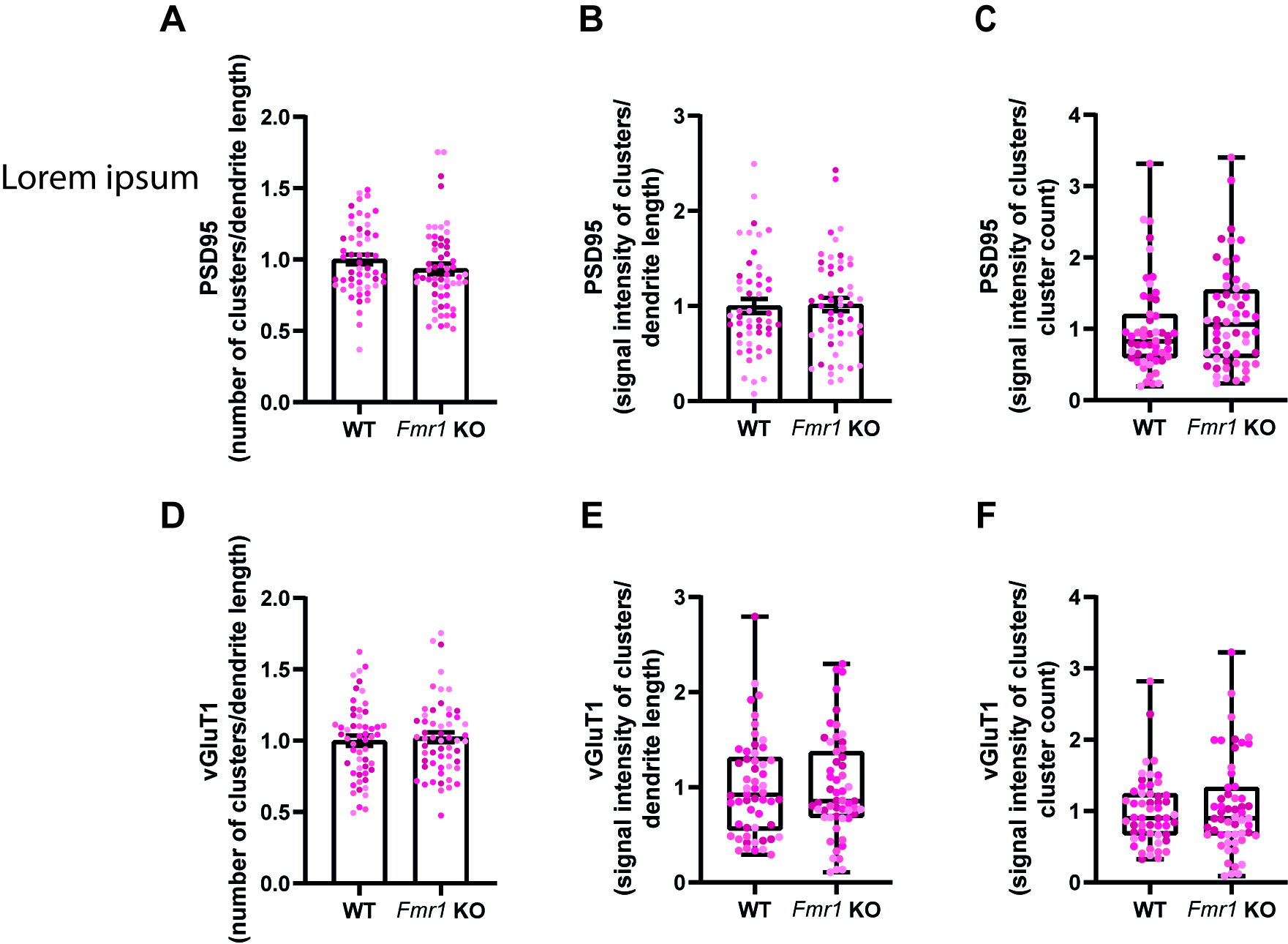

Supplement: Supplementary file 1 — Supplementary Figure S1 (TIF 10281 KB) [file 12031_2025_2325_MOESM1_ESM.tif]
