# Supplementary material for: FMRP Controls Neuronal Architecture and Synaptic Content of NMDA Receptors in Cultured Hippocampal Neurons
Source: J Mol Neurosci. 2025 Apr 2;75(2):44. doi: 10.1007/s12031-025-02325-8 (PMC11965214; doi:10.1007/s12031-025-02325-8)
Supplement: Supplementary file 2 — Supplementary Table 1 (DOCX 18 KB) [file 12031_2025_2325_MOESM2_ESM.docx]

**Table I: List and reference of the antibodies used in this work**

| Antibody | Species | Dilution | Catalogue number | Brand |
| --- | --- | --- | --- | --- |
| Anti-GluA1 | Rabbit | 1:100 | AGC_004 | Alomone |
| Anti-GluA2 | Rabbit | 1:100 | AGC_005 | Alomone |
| Anti-GluN2A | Rabbit | 1:100 | AGC_002 | Alomone |
| Anti-GluN2B | Rabbit | 1:100 | AGC_003 | Alomone |
| Anti-MAP2 | Chicken | 1:10000 | Ab5392 | Abcam |
| Anti-PSD95 | Mouse | 1:200 | 7E3_1B8 | Thermo Scientific |
| Anti-vGluT1 | Guinea Pig | 1:5000 | AB5905 | Millipore |
| Anti-AnkG | Mouse | 1:500 | MABN466 | Millipore |
| Alexa Fluor 405 – conjugated anti-chicken | Goat | 1:500 | A-48260 | Invitrogen |
| Alexa Fluor 488 – conjugated anti-mouse | Goat | 1:500 | A-11001 | Invitrogen |
| Alexa Fluor 568 – conjugated anti-rabbit | Goat | 1:500 | A-11036 | Invitrogen |
| Alexa Fluor 647 – conjugated anti-guinea pig | Goat | 1:500 | A-21450 | Invitrogen |
